# Radiographic Findings Concerning for Gangrenous Cholecystitis in an Asymptomatic Elderly Patient: A Case for Conservative Management

**DOI:** 10.7759/cureus.116442

**Published:** 2026-09-17

**Authors:** Zoya Syed, Mahad Azhar, James Gervasoni

**Affiliations:** 1 School of Medicine, St. George's University School of Medicine, St. George's, GRD; 2 General Surgery/Surgical Oncology, Saint Peter’s University Hospital, New Brunswick, USA

**Keywords:** acute cholecystitis, atypical presentation of gangrenous cholecystitis, chole, cholecystectomy, elderly surgery, gall bladder diseases and gallstones, gallbladder necrosis, gangrenous cholecystitis, laparoscopic chole, radiographic findings

## Abstract

Gallbladder necrosis is a rare but life-threatening complication of acute cholecystitis, likely resulting from chronic blood flow, inflammation, and intraluminal pressure. It most commonly occurs in elderly or critically ill patients with significant comorbidities and is often underreported due to its nonspecific presentation. This diagnosis is usually associated with right upper quadrant pain, leukocytosis, and fever. Here, we present an 80-year-old woman with metastatic lung cancer, COPD, and an extensive surgical and oncologic history who was incidentally found to have radiographic findings concerning for gangrenous cholecystitis but remained asymptomatic. Despite these findings, the patient remained clinically stable with improving leukocytosis during her inpatient course and was managed conservatively without surgical intervention. Post-discharge follow-up was unavailable.

## Introduction

Gallbladder necrosis is a rare but serious sequela of acute cholecystitis resulting from vascular compromise of the gallbladder wall due to chronic inflammation and increased intraluminal pressure. Although uncommon, it is associated with significant morbidity and mortality, particularly in elderly and immunocompromised individuals. Classic clinical signs of necrosis include fever, leukocytosis, and right upper quadrant pain, but presentations may vary significantly in patients with multiple comorbidities [1,2]. In such cases, diagnosis often relies on radiographic findings such as gallbladder distention, mucosal sloughing, and pericholecystic fluid, raising suspicion for tissue compromise [3]. Despite these red flags, the decision to pursue surgical intervention remains challenging since the patient is clinically stable and asymptomatic [4-7]. Conservative management in suspected gangrenous cholecystitis remains controversial because delaying source control may allow progression to perforation, sepsis, and other life-threatening complications. Conversely, operative intervention may carry substantial morbidity in elderly patients with significant comorbidities, creating a difficult balance between the risks of untreated disease and those of invasive treatment. This report contributes to the limited literature on atypical presentations of gallbladder necrosis and highlights the importance of tailoring management to the individual risk profile and clinical context of complex patients.

## Case presentation

An 80-year-old female with an extensive past medical history - including 40 pack-years of smoking, COPD, anemia, bilateral vocal cord paralysis, bilateral breast cancer status post-surgery, chemotherapy, and radiation, left lung cancer (status post-VATS lobectomy and chemotherapy), right lung cancer (status post chemotherapy), and mediastinal adenopathy positive for adenocarcinoma - presented from a subacute rehabilitation center after sustaining two hip fractures. Her oncologic history also includes metastatic lung cancer with bone metastases status post radiotherapy to the right hip and iliac, right femur peritrochanteric lesions status post prophylactic right hemiarthroplasty, and left hip fracture status post arthroplasty. The patient also had a chronically elevated total bilirubin for one year, thought to be secondary to Gilbert syndrome.

On physical exam, the patient's temperature was afebrile (98.3°F), with a pulse of 61 bpm, blood pressure of 133/53 mmHg, and oxygen saturation of 100% on a 3 L nasal cannula. She appeared cachectic without abdominal pain or distress. Physical examination was positive for hoarseness and bradycardia in the 50s. Examination of the abdomen was benign and revealed a soft, non-distended abdomen without tenderness, rebound, or guarding.

Her lab values were significant for leukocytosis (WBC of 15.3), anemia (HGB 7.6), total bilirubin 1.4 mg/dL, alkaline phosphatase 70 U/L, AST 27 U/L, and ALT 16 U/L (Table 1).

**Table 1:** Laboratory findings demonstrating leukocytosis, anemia, and mild hyperbilirubinemia at presentation, with preserved liver enzyme levels. Follow-up testing demonstrated improvement in leukocytosis. Reference ranges are provided for clinical interpretation.

Laboratory Test	Patient Value	Reference Range	Interpretation
White blood cell count (WBC)	15.3 × 10³/µL	4.0–11.0 × 10³/µL	Elevated (leukocytosis)
Hemoglobin (Hgb)	7.6 g/dL	12.0–16.0 g/dL	Low (anemia)
Total bilirubin	1.4 mg/dL	0.2–1.2 mg/dL	Mildly elevated
Alkaline phosphatase (ALP)	70 U/L	44–147 U/L	Within normal limits
Aspartate aminotransferase (AST)	27 U/L	10–40 U/L	Within normal limits
Alanine aminotransferase (ALT)	16 U/L	7–56 U/L	Within normal limits
WBC (follow-up)	13.0 × 10³/µL	4.0–11.0 × 10³/µL	Elevated, but improved from presentation

Right upper quadrant ultrasound indicated gallstones, gallbladder wall thickening, sloughed mucosal membranes, and pericholecystic fluid, concerning for gallbladder necrosis (Figures 1, 2). A computed tomography (CT) with IV contrast of the abdomen showed gallbladder distension, hyperdensity suggesting sludge/stones, mild pericholecystic infiltration suggestive of cholecystitis, moderate right-sided pleural effusion, and aortic atherosclerosis (Figure 3). No intramural or intraluminal gas was identified to suggest emphysematous cholecystitis, and there was no focal wall defect or other radiographic evidence of gallbladder perforation. Additional inflammatory markers, including C-reactive protein and procalcitonin, were not obtained, and a formal geriatric frailty assessment was not documented. Serial laboratory evaluation demonstrated improvement in leukocytosis, with the WBC decreasing from 15.3 to 13.0 × 10³/µL.

**Figure 1:**
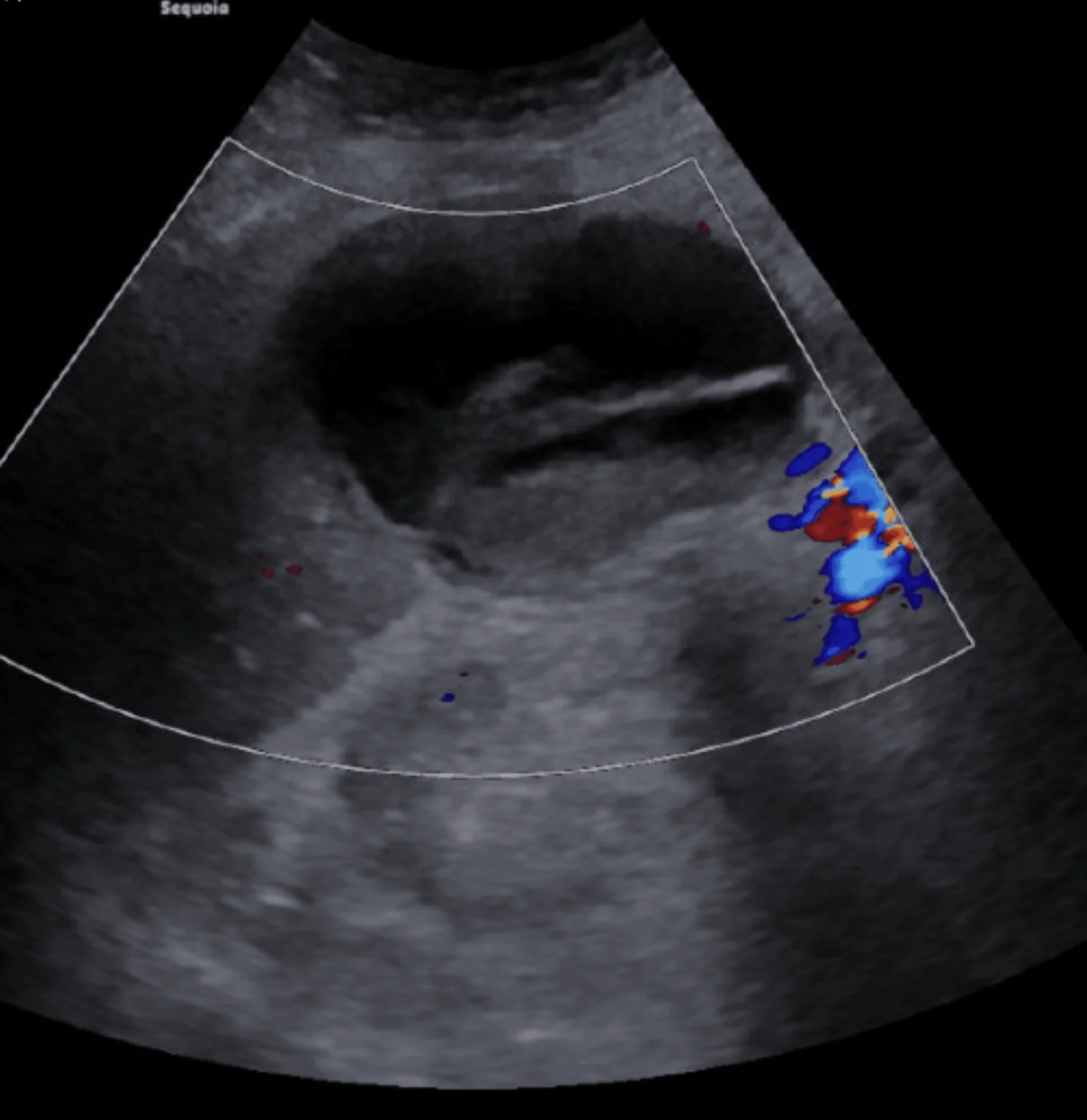
Right upper quadrant ultrasound demonstrating cholelithiasis, gallbladder wall thickening, and pericholecystic fluid, concerning for gallbladder necrosis.

**Figure 2:**
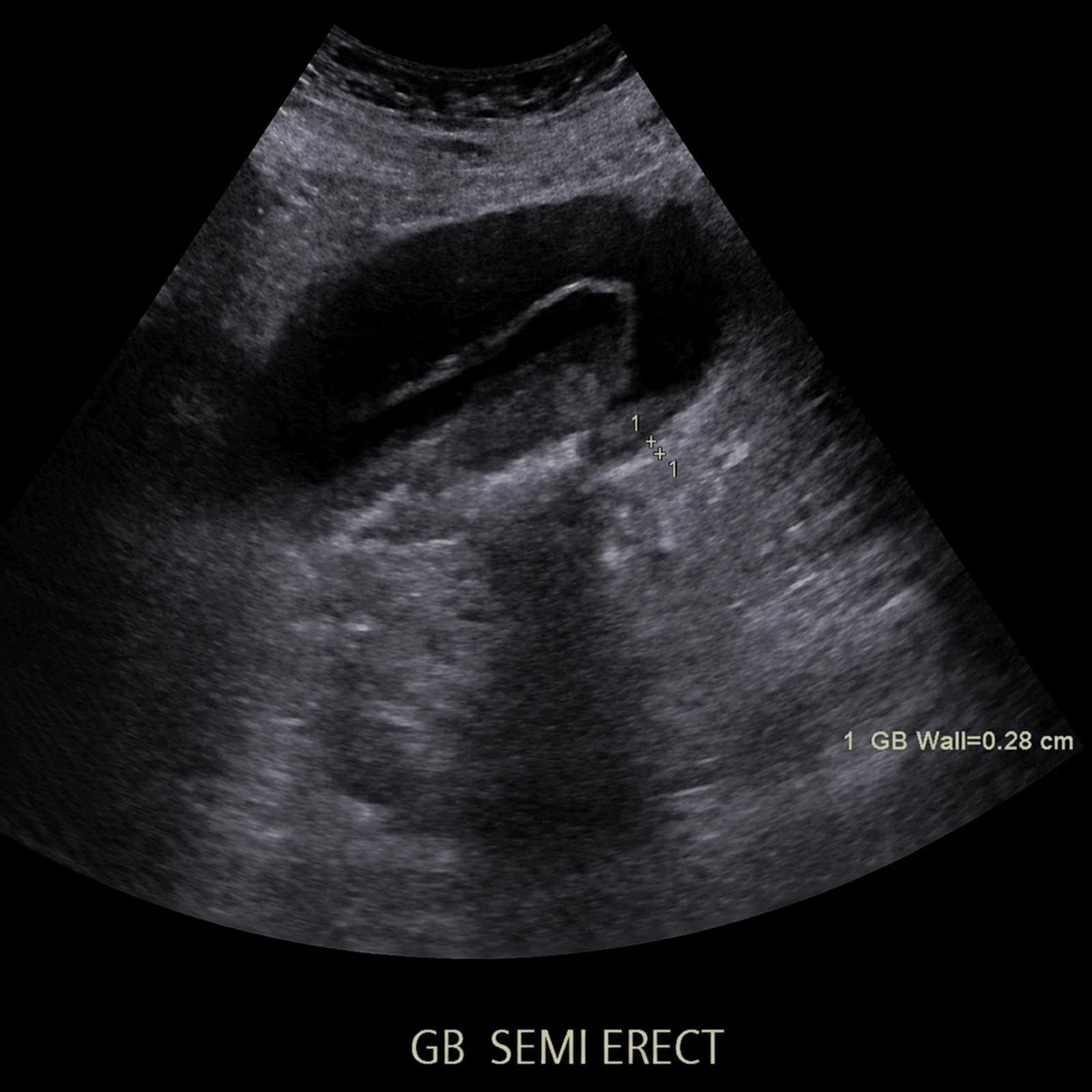
Right upper quadrant ultrasound of the gallbladder demonstrating wall thickening with sloughed mucosal membranes and pericholecystic fluid, findings concerning for gallbladder necrosis.

**Figure 3:**
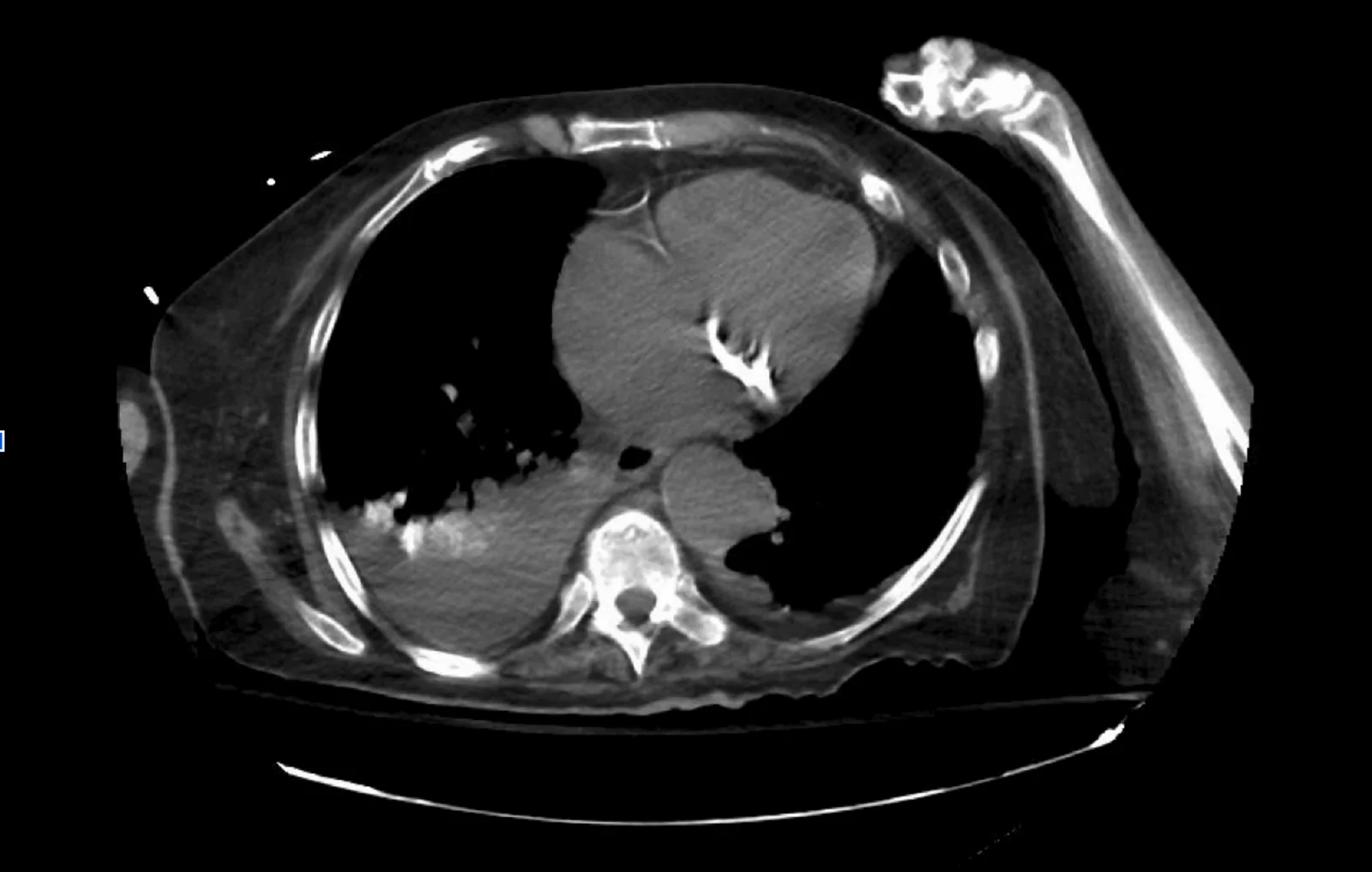
Contrast-enhanced CT of the abdomen demonstrating gallbladder distension with hyperdense intraluminal sludge/stones and mild pericholecystic inflammatory changes.

Initial management included IV antibiotics and consideration for percutaneous cholecystostomy. Details regarding the specific antibiotic regimen and duration were not available. Clinical monitoring consisted of serial physical examinations and laboratory assessment, with continued observation of oral intake and hemodynamic stability. However, due to clinical stability, improving leukocytosis (WBC decreasing to 13.0), and tolerance of an oral diet, suspicion for acute cholecystitis decreased and conservative management continued without surgical intervention. Post-discharge follow-up data were not available.

## Discussion

Gallstones, the leading cause of acute cholecystitis, are highly prevalent worldwide. Although the majority of patients with gallstones remain asymptomatic, a subset develops symptomatic disease. Acute cholecystitis requires prompt evaluation and treatment because of the risk of progression to complications such as gangrenous cholecystitis and gallbladder perforation.

Gallbladder necrosis is a recognized complication of acute cholecystitis and is most commonly seen in elderly, immunocompromised, or critically ill patients. Although underreported, studies estimate gangrenous or necrotic changes to occur in 15-25% of acute cholecystitis cases [1]. The pathophysiology involves inflammation and increased intraluminal pressure leading to compromised blood supply and ischemia of the gallbladder wall, which can ultimately progress to necrosis and perforation. Risk factors include advanced age, delayed surgical intervention, cardiovascular disease, and immunosuppression, including diabetes mellitus, all of which may impair tissue oxygenation and promote inflammation [2]. Several of these risk factors were relevant to this patient, who was elderly and had an extensive history of metastatic cancer, COPD, and radiation exposure.

Radiologic evaluation is important when gallbladder necrosis is suspected, particularly in patients with atypical clinical presentations. CT findings associated with gangrenous changes include intraluminal membranes resulting from sloughed mucosa, gallbladder wall thickening, pericholecystic fluid, lack of mucosal enhancement, and gas within the gallbladder wall or lumen. Similarly, ultrasound findings associated with gallbladder necrosis include wall thickening, sloughed membranes, and pericholecystic fluid [3]. However, these findings must be interpreted within the clinical context because imaging alone cannot reliably distinguish chronic inflammatory changes from true necrosis. In this patient, the absence of intramural or intraluminal gas, focal wall defects, or other radiographic evidence of perforation further limited the ability to establish a definitive diagnosis. Some of the observed radiographic abnormalities could also have reflected chronic inflammatory changes rather than true necrosis, contributing to the diagnostic uncertainty.

Management becomes less straightforward when concerning radiographic findings occur in a clinically stable patient with minimal or no symptoms. Surgical intervention with laparoscopic cholecystectomy is the mainstay treatment for suspected necrosis, while percutaneous drainage may be considered in unstable or high-risk patients, as outlined by the 2018 Tokyo Guidelines (TGs) [4]. The TGs classify biliary infections as mild, moderate, or severe based on local and systemic signs of inflammation, imaging findings, and the presence of organ dysfunction [4]. In this patient, application of this framework was complicated by discordance between the radiographic and clinical findings. Although imaging raised concern for complicated cholecystitis and the patient had leukocytosis, she remained afebrile and hemodynamically stable without abdominal pain, tenderness, or organ dysfunction.

Although her leukocytosis improved from 15.3 to 13.0 × 10³/µL, it remained elevated and was therefore interpreted as a nonspecific inflammatory marker rather than evidence of resolution of gallbladder disease. She continued to tolerate an oral diet. The patient's mild hyperbilirubinemia was chronic and had previously been attributed to Gilbert syndrome; however, its etiology could not be definitively established in this case. Given the normal alkaline phosphatase and transaminase levels, the isolated mild bilirubin elevation was not considered specific evidence of acute biliary obstruction. Taken together with her clinical stability and substantial comorbidity burden, these findings contributed to weighing the risks and benefits of invasive source control against continued close observation. This case therefore illustrates the importance of applying guideline-based recommendations within the context of the individual patient's clinical course and operative risk. Clinical judgment continues to play a crucial role in the management of medically complex patients.

Importantly, this case should not be interpreted as evidence against urgent source control when gangrenous cholecystitis or gallbladder perforation is strongly suspected or confirmed. Rather, it highlights the uncertainty that may arise when concerning radiographic findings are discordant with an otherwise stable clinical presentation. Because the diagnosis in this patient was based on radiographic suspicion without surgical or histopathologic confirmation, improvement in clinical status and leukocytosis cannot be interpreted as confirmation of resolution of gallbladder necrosis. This case adds to the literature on atypical presentations of suspected gallbladder necrosis and emphasizes the importance of individualized risk assessment. Further research is needed to better define which high-risk patients with discordant clinical and radiographic findings may be appropriate candidates for nonoperative management. Careful physical examination, clinical judgment, and close monitoring remain essential when determining the appropriate management strategy.

## Conclusions

Gallbladder necrosis remains a serious complication of acute cholecystitis, particularly in elderly and medically complex patients. While concerning radiographic findings warrant careful evaluation, management decisions should incorporate the patient’s clinical stability, physical examination, laboratory trends, comorbidities, and procedural risk rather than imaging findings alone. In high-risk patients with discordant clinical and radiographic findings, individualized risk assessment and close monitoring are essential when determining the appropriate management strategy. Importantly, this approach should not delay urgent source control when gangrenous cholecystitis or perforation is strongly suspected or confirmed.

This report underscores the value of an individualized approach considering radiographic suspicions and patient factors such as comorbidities. Clinicians must take caution not to over-rely on imaging findings alone. Further refinement of the current criteria is needed to define which patients may benefit from nonoperative versus operative management.
